# A *Narnavirus*-Like Element from the Trypanosomatid Protozoan Parasite *Leptomonas seymouri*

**DOI:** 10.1128/genomeA.00713-16

**Published:** 2016-08-04

**Authors:** Lon-Fye Lye, Natalia S. Akopyants, Deborah E. Dobson, Stephen M. Beverley

**Affiliations:** Department of Molecular Microbiology, Washington University School of Medicine, St. Louis, Missouri, USA

## Abstract

Genome sequences were determined for a novel RNA virus, *Leptomonas seymouri* Narna-like virus 1 (LepseyNLV1). A 2.9-kb segment encodes an RNA-dependent RNA polymerase (RdRp), while a smaller 1.5-kb segment showed no database search matches. This is the first report of bisegmented *Narnaviridae* from insect trypanosomatids.

## GENOME ANNOUNCEMENT

From *in silico* screens of metatranscriptomic datasets for protozoan viruses, we encountered within archives of *Leptomonas seymouri* (order *Kinetoplastida*, Excavata eukaryotic supergroup) a hit to members of the *Narnaviridae* (1–3*)*, which had not been previously identified in trypanosomatid protozoa*.* From a total of 300 million reads, 0.22% were assembled into a contig of 2,914 nucleotides (nt), predicting a 932-amino acid (aa) protein that bore motifs typical of narnaviral RNA-dependent RNA polymerase (RdRps) (1–3). As this was absent from previous assemblies (4), we isolated RNA from the *L. seymouri* strain ATCC 30220 using TRIzol reagent (Thermo Fisher), followed by digestion with DNase I (Thermo Fisher), purification with RCC-25 kit (Zymo Research), and digestion by S1 nuclease (Thermo Fisher).

Two cytoplasmic double-stranded RNAs (dsRNAs) of ~3 and ~1.5 kb were visualized after agarose gel electrophoresis (4, 5), which were eluted and used for generation of cDNA. Reverse transcriptase-PCR (RT-PCR) tests showed that the 3-kb band corresponded to the 2,914-nt RdRp contig. cDNA for the 1.5-kb band was inserted into bacterial vectors and sequenced (6), identifying a 1,455-nt contig in the metatranscriptomic assembly, which was confirmed by RT-PCR amplification. Blast-based searches did not yield any matches in the sequence databases tested.

Provisionally, we term these elements the *L. seymouri* Narna-like virus 1 (LepseyNLV1) L and S segments; however, their functional association remains to be proven. Some data suggest that both segments are unstable during culture, as sensitive RT-PCR tests of this strain acquired independently from another laboratory did not reveal them (the authenticity of this strain was confirmed by sequence of two nuclear genes, *GAPDH* and *PTR1*). As in other multisegmented RNA viruses, loss of the RdRp would be expected to result in the loss of the remaining segments.

Viruses in the family *Narnaviridae* (“naked RNA”) lack capsids or envelopes and do not form infectious viral particles. They reside in the cytosol as an RNA-protein complex containing one single-stranded RNA segment of about 3 kb, with a single open reading frame (ORF) encoding the RdRp (1–3). Two genera are recognized; unlike cytosolic narnaviruses, mitoviruses are found in the mitochondrion of fungi and translated using the mitochondrial genetic code (2). Phylogenetic comparisons of the L segment RdRp with other *Narnaviridae* place it firmly within *Narnavirus* as a new species, since the overall amino acid divergence exceeds 80%. Interestingly, the trisegmented *Ourmiavirus* spp. show a closer relationship to *Narnavirus* than to *Mitovirus* (2, 7), suggesting that the bisegmented LepseyNLV1 exhibits some characteristics intermediate between *Narnavirus* and *Ourmiavirus*.

*Leptomonas* is a monoxenous kinetoplastid parasite of insects, and related parasites are widespread in insects around the world (8). Interestingly, *L. seymouri* has been repeatedly isolated from patients infected by *Leishmania donovani* (9), although *in vitro*, it is unable to survive in mammalian macrophages (4, 10). Because viruses within the related protozoan *Leishmania guyanensis* have been associated with elevated pathogenicity in animal models (11), LepseyNLV1 potentially contributes to the pathogenicity of *Leptomonas-Leishmania* coinfections (9).

### Nucleotide sequence accession numbers.

The genome sequences of the LepseyNLV1 L and S segments have been deposited in GenBank under accession numbers KU935604 and KU935605.
